# Study of sulfoglycolysis in *Enterococcus gilvus* reveals a widespread bifurcated pathway for dihydroxypropanesulfonate degradation

**DOI:** 10.1016/j.isci.2024.111010

**Published:** 2024-09-21

**Authors:** Yiwei Chen, Ruoxing Chu, Kailiang Ma, Li Jiang, Qiaoyu Yang, Zhi Li, Min Hu, Qiuyi Guo, Fengxia Lu, Yifeng Wei, Yan Zhang, Yang Tong

**Affiliations:** 1 College of Food Science and Technology, Nanjing Agricultural University, Nanjing 210095, China; 2 New Cornerstone Science Laboratory, School of Pharmaceutical Science and Technology, Tianjin University, Tianjin 300072, China; 3 Tianjin Key Laboratory for Modern Drug Delivery & High-Efficiency, Collaborative Innovation Center of Chemical Science and Engineering, School of Pharmaceutical Science and Technology, Tianjin University, Tianjin 300072, China; 4 Singapore Institute of Food and Biotechnology Innovation, Agency for Science, Technology and Research (A∗STAR), Singapore 138669, Singapore; 5 Frontiers Science Center for Synthetic Biology (Ministry of Education), Tianjin University, Tianjin 300072, China; 6 Key Laboratory of Systems Bioengineering (Ministry of Education), School of Chemical Engineering and Technology, Tianjin University, Tianjin 300072, China; 7 School of Life Sciences and Biotechnology, Shanghai Jiao Tong University, Shanghai 200240, China; 8 Carbon-Negative Synthetic Biology for Biomaterial Production from CO2 (CNSB), Campus for Research Excellence and Technological Enterprise (CREATE), 1 CREATE Way, Singapore 138602, Singapore

**Keywords:** Biological sciences, Microbiology, Microbial metabolism

## Abstract

Sulfoquinovose (SQ), the polar head group of sulfolipids essential for photosynthesis, is naturally abundant. Anaerobic Firmicutes degrade SQ through a transaldolase-dependent (sulfo-TAL) pathway, producing dihydroxypropanesulfonate (DHPS). Some bacteria extend this pathway by the sequential action of HpfG and HpfD converting DHPS to 3-hydroxypropanesulfonate (3-HPS) via 3-sulfopropionaldehyde (3-SPA). Here, we report a variant sulfo-TAL pathway in *Enterococcus gilvus*, involving additional enzymes, a NAD^+^-dependent 3-SPA dehydrogenase HpfX, and a 3-sulfopropionyl-CoA synthetase HpfYZ, which oxidize 3-SPA to 3-sulfopropionate (3-SP) coupled with ATP formation. *E. gilvus* grown on SQ or DHPS produced a mixture of 3-HPS and 3-SP, indicating the bifurcated pathway. Similar genes are found in various Firmicutes, including gut bacteria. Importantly, 3-SP, but not 3-HPS, can serve as a respiratory terminal electron acceptor for *Bilophila wadsworthia*, a common intestinal pathobiont, resulting in the production of toxic H_2_S. This research expands our understanding of sulfonate metabolism and reveals cross-feeding in the anaerobic microbiome.

## Highlights

• An extended sulfo-TAL pathway involving HpfXYZ was identified in *E. gilvus*
• HpfXYZ were biochemically characterized
• The pathway enables disproportionation of environmental DHPS into 3-HPS and 3-SP
• These sulfonate conversions affect the growth of human disease-linked SSRB

## Graphical abstract

**Figure:**
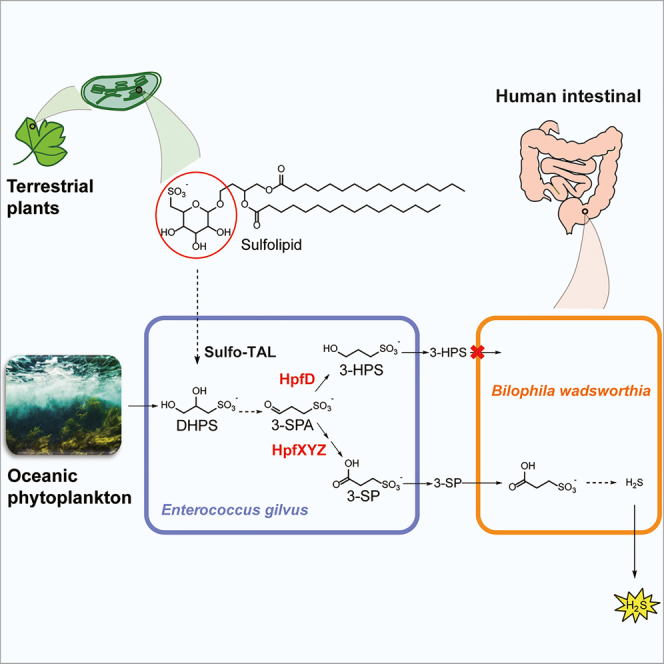

## Introduction

Sulfoquinovose (SQ) is a sulfonated glucose derivative that serves as the polar head group of sulfoquinovosyl diacylglycerol (SQDG) in thylakoid membranes of chloroplasts in plants, algae, and other photosynthetic eukaryotes, as well as many phototrophic bacteria.^1^^,^^2^ As a major biological organosulfur compound, SQ’s global annual production is estimated at 10^10^ tons.^3^ Its utilization as a growth substrate has been observed in bacteria across diverse environments, and recent studies have shed light on the molecular intricacies of SQ metabolism in various bacterial species.^4^^,^^5^ Bacterial SQ metabolism is initiated through the release of SQ from SQ-glycerol, catalyzed by the sulfoquinovosidase YihQ^6^ or SqgA.^7^ The structural similarity of SQ to glucose-6-phosphate led to the proposal of a glycolytic pathway for its degradation, termed sulfoglycolysis.^1^ To date, four distinct sulfoglycolytic pathways have been identified: one resembling the Embden-Meyerhof-Parnas pathway (sulfo-EMP), one similar to the Entner-Doudoroff pathway (sulfo-ED), and two more relying on either a transaldolase (sulfo-TAL) or a transketolase (sulfo-TK) closely related to enzymes in the pentose phosphate pathway.^5^ In addition, two non-sulfoglycolytic SQ degradation pathways have been reported, involving oxygenolytic desulfonation of SQ to form 6-deoxyglucose, and reduction to glucose, which is then metabolized via standard glycolytic pathways.^8^^,^^9^^,^^10^

The four sulfoglycolytic pathways do not entail cleavage of the sulfonate C-S bond, and lead to the generation of various sulfonate byproducts. The sulfo-EMP,^9^^,^^11^ sulfo-ED,^12^ and sulfo-TAL^13^^,^^14^ pathways enable the use of three of the SQ carbons and generates sulfolactaldehyde (SLA) as a key intermediate, which is either reduced by SLA reductase YihU to dihydroxypropanesulfonate (DHPS)^15^ or oxidized by SLA dehydrogenase SlaB to sulfolactate as a byproduct. Meanwhile, the sulfo-TK pathway utilizes four of the SQ carbons and generates sulfoacetaldehyde as an intermediate, which is converted to either isethionate or sulfoacetate as a byproduct.^9^^,^^16^ The resulting C3 and C2 sulfonates are further degraded by other bacteria, which release the sulfonate sulfur as either SO_3_^2−^, SO_4_^2−^, or H_2_S.^17^^,^^18^ SQ degradation and sulfonate sulfur mineralization by environmental bacteria play an integral role in the biological sulfur cycle.^19^

Bacterial SQ metabolism is of particular interest in the human digestive system, where consortia of anaerobic gut bacteria degrade dietary SQ to produce H_2_S, with implications for intestinal health.^20^ Among the gut bacteria studied, Gram-negative fermenting bacteria in the family Enterobacteriaceae, including *Escherichia coli*,^11^ *Klebsiella*^21^ and *Citrobacter* sp.^22^ degrade SQ via the sulfo-EMP pathway. Meanwhile, the more highly abundant Gram-positive fermenting bacteria in the phylum Firmicutes, including *Enterococcus gilvus*, *Clostridium symbiosum*, and *Eubacterium rectale*^13^^,^^23^ degrade SQ via the sulfo-TAL pathway. Both pathways produce DHPS, which is further metabolized by sulfate- and sulfite-reducing bacteria (SSRB).^22^^,^^23^^,^^24^ These bacteria cleave off the sulfonate group to release sulfite for use as a terminal electron acceptor (TEA), generating H_2_S. A notable human gut SSRB is *Bilophila wadsworthia*, a widespread pathobiont.^25^^,^^26^ *B. wadsworthia* is unable to carry out sulfate reduction, but obtains sulfite required as a TEA from the degradation of various sulfonates, including taurine^26^ and the sulfoglycolytic products DHPS,^27^ sulfolactate,^22^ isethionate^28^^,^^29^ and sulfoacetate.^30^ Apart from SSRB, DHPS is also metabolized by certain fermenting bacteria, which convert it to 3-HPS.^27^ In this pathway, DHPS is converted to 3-sulfopropionaldehyde (3-SPA) by the oxygen-sensitive glycyl radical enzyme DHPS dehydratase HpfG (and its activating enzyme HpfH), followed by reduction to 3-HPS by the NADH-dependent sulfopropionaldehyde reductase HpfD, and 3-HPS export by the transporter HpfE.^27^ HpfDEGH allow these fermenting bacteria to utilize DHPS as an NADH sink. In some bacteria, these enzymes are associated with sulfo-EMP or sulfo-TAL genes, forming an extended sulfoglycolysis pathways with 3-HPS as the end-product.^5^ The identity of the SQ-derived sulfonate product determines its subsequent metabolism by different SSRB, affecting H_2_S production in the microbiome.

A recent study in our laboratory identified a variant of the sulfo-TK pathway in fermenting bacteria generating sulfoacetate as a byproduct.^16^ In this pathway, the sulfoacetaldehyde intermediate is oxidized to sulfoacetate by the sequential action of a CoA-acylating sulfoacetaldehyde dehydrogenase (SqwD), followed by an ADP-forming sulfoacetate-CoA ligase (SqwKL), coupled to the phosphorylation of ADP to ATP.^16^ The observation that some sulfoglycolytic pathway variants yield C1-alcohols (DHPS, isethionate), while others yield the corresponding carboxylic acids (sulfolactate, sulfoacetate), led us to consider the possibility of a HpfG-dependent extended sulfoglycolytic pathway generating 3-sulfopropionate (3-SP) as a byproduct. While reviewing SQ degradation gene clusters, we previously noted that the sulfo-TAL gene cluster in *E. gilvus* is associated with HpfG and HpfD, as well as a homolog of succinic semialdehyde dehydrogenase and ADP-forming succinyl-CoA synthetase, suggesting a mechanism for formation of 3-SP in addition to 3-HPS.^5^ Although SQ degradation by *E. gilvus* was previously studied,^13^ the formation of 3-HPS and 3-SP as sulfoglycolytic byproducts has not been investigated.

In this study, we examine the HpfG-dependent extended sulfo-TAL pathway in *E. gilvus* ATCC BAA-350/DSM 15689, originally isolated from the bile of a patient with cholecystitis,^31^ and confirm the formation of both 3-HPS and 3-SP as byproducts. In this pathway, the 3-SPA intermediate is either reduced to 3-HPS by HpfD, or oxidized to 3-SP by the sequential action of a CoA-acylating 3-sulfopropionaldehyde dehydrogenase (HpfX) and an ADP-forming 3-sulfopropionate-CoA ligase (HpfYZ), coupled to the phosphorylation of ADP to ATP. We also report a notable finding that 3-SP but not 3-HPS can be utilized by *B. wadsworthia* as a source of TEA for its respiratory growth, forming H_2_S. Gene clusters encoding HpfXYZ in association with HpfG and HpfD occur in various Firmicutes bacteria, suggesting the widespread occurrence of the bifurcated DHPS degradation pathway.

## Results

### Genes in the extended sulfo-TAL gene cluster of *E. gilvus* ATCC BAA-350

**Figure 1:**
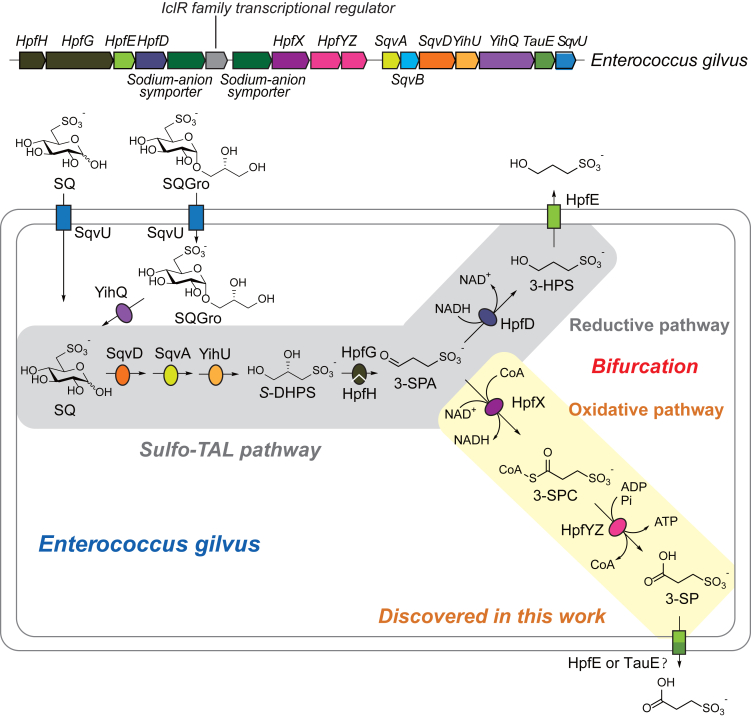
A variant extended sulfo-TAL gene cluster and pathway in *E. gilvus* SqvU (Uniprot: R2VCK9), sugar transporter; YihQ (Uniprot: R2XZF3), sulfoquinovosidase; SqvD (Uniprot: R2XKF3), SQ isomerase; SqvA (Uniprot: R2VCR4), SF transaldolase; YihU (Uniprot: R2XLK5), SLA reductase; HpfG (Uniprot: R2VCM5), DHPS dehydratase; HpfH (Uniprot: R2VCS2), activating enzyme for HpfG; HpfD (Uniprot: R2XLL6), 3-sulfopropionaldehyde reductase; HpfE (Uniprot: R2XKG4), 3-HPS exporter; HpfX (Uniprot: R2XKF7), 3-sulfopropionaldehyde dehydrogenase; HpfYZ (Uniprot: R2XLL0, R2XZF8), 3-sulfopropionate-CoA ligase; TauE (Uniprot: R2VCQ9), probable 3-sulfopropionate exporter; SQ, sulfoquinovose; SQGro, sulfoquinovosyl glycerol; SF, 6-deoxy-6-sulfofructose; SLA, sulfolactaldehyde; DHPS, dihydroxypropanesulfonate; 3-SPA, 3-sulfopropionaldehyde; 3-HPS, 3-hydroxypropanesulfonate; 3-SPC, 3-sulfopropionyl-CoA; 3-SP, 3-sulfopropionate. The abbreviations of proteins and substrates in this study were listed in Table S2.

The sulfo-TAL pathway involves conversion of SQ to 6-deoxy-6-sulfofructose (SF) by SQ isomerase (SqvD), and sulfo-sugar cleavage by a transaldolase (SqvA),^32^ with glyceraldehyde 3-phosphate (G3P), G3P as the ketol acceptor to release SLA.^13^^,^^14^ In the aerobic *Bacillus megaterium*^14^ and *Bacillus aryabhattai*,^13^ SLA is oxidized to sulfolactate by an aldehyde dehydrogenase (SlaB), while in the anaerobic intestinal bacteria *Clostridium symbiosum* and *Eubacterium rectale*,^13^ SLA is reduced to DHPS by SLA reductase (YihU). In *E. gilvus*, the genome neighborhood of the sulfo-TAL gene cluster contains HpfGHDE, consistent with an extended sulfo-TAL pathway forming 3-HPS.^5^ In addition, the genome neighborhood also contains a homolog of aldehyde dehydrogenase (HpfX, Pfam: PF00171) and ADP-forming succinate-CoA ligase (HpfYZ, Pfam: PF08442 and PF02629). We hypothesized that HpfX and HpfYZ act sequentially to oxidize 3-SPA to 3-sulfopropionyl-CoA (3-SPC), followed by conversion to 3-SP by 3-sulfopropionate-CoA ligase (HpfYZ), coupled to the phosphorylation of ADP to ATP (Figure 1).^5^ This would lead to a bifurcated extended sulfo-TAL pathway, with the production of both 3-HPS and 3-SP.

To facilitate biochemical experiments, we recombinantly produced and purified HpfD (*Eg*HpfD, Uniprot: R2XLL6) and HpfX (*Eg*HpfX, Uniprot: R2XKF7) (Figures S1A and S1B). N-terminal His_6_-tagged HpfY (*Eg*HpfY, Uniprot: R2XLL0) and untagged HpfZ (*Eg*HpfZ, Uniprot: R2XZF8) were co-expressed followed by Ni^2+^ affinity chromatography, leading to co-purification of the two subunits in a 1:1 molar ratio as quantified by densitometric analysis of Coomassie Blue-stained SDS-PAGE bands (Figure S1C).

### Activity assays of HpfD

The physiological reaction catalyzed by HpfD is the NADH-dependent reduction of 3-SPA to 3-HPS, which is thermodynamically reversible. *In vitro*, HpfD also catalyzes NAD^+^-dependent oxidation of 3-HPS. The activity of *E. gilvus* HpfD was detected by incubation of purified HpfD with 3-HPS and NAD^+^. A time-dependent increase in absorbance at 340 nm (A_340 nm_) was observed, indicating the oxidation of 3-HPS accompanied by formation of NADH. The optimal pH for this reaction was 9.5 (Figure S2A). Activity was proportional to the amount of HpfD added (Figure S2B), and no reaction was observed in negative controls where the enzyme or one of the substrates was omitted. The apparent Michaelis-Menten kinetic parameters of HpfD were determined with 0.1 μM HpfD, by varying the concentrations of one substrate while maintaining the other in excess (*k*_cat_ = 15.30 ± 0.37 s^−1^, *K*_M_ = 4.97 ± 0.57 mM for 3-HPS; *k*_cat_ = 20.87 ± 0.84 s^−1^, *K*_M_ = 0.64 ± 0.09 mM for NAD^+^) (Figures S2C and S2D).

**Figure 2:**
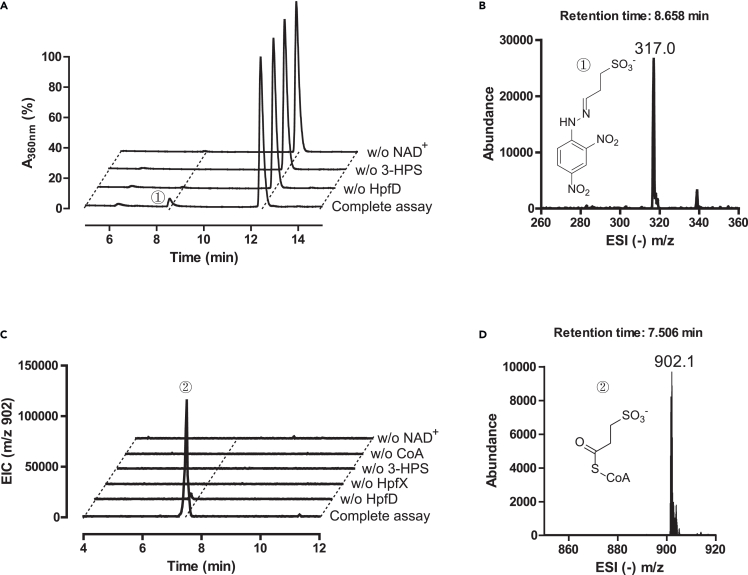
Activity assays of HpfD and HpfX (A) Elution profiles of DNPH-derivatized aldehyde product, formed by HpfD-catalyzed oxidation of 3-HPS, monitoring absorbance at 360 nm. (B) Negative ionization mass spectra of DNPH-derivatized reaction product, corresponding to peak 1 in a). (C) HPLC elution profiles of the products of HpfD-HpfX coupling reaction, monitoring extracted ion counts of ESI negative mode. (D) Negative ionization mass spectrum of peak 2 in c), corresponding to the reaction product 3-SPC.

The reaction product 3-SPA underwent derivatization with 2,4-dinitrophenylhydrazine (DNPH), followed by liquid chromatography-mass spectrometry (LC-MS) analysis. A prominent A_360 nm_ peak in the elution profile corresponding to an electrospray ionization negative mode (ESI (−)) *m*/*z* ratio of 317.0 is consistent with the formation of 3-SPA-DNPH in the full reaction mixture. Conversely, this peak was absent in the negative controls where either HpfD, 3-HPS, or NAD^+^ was excluded (Figures 2A and 2B).

### LC-MS assay for HpfD-HpfX reaction

The ability of the HpfX to oxidize 3-SPA into 3-SPC was ascertained by incubating the purified enzymes (HpfD and HpfX) with 3-HPS, NAD^+^ and CoA, followed by analysis of the reaction product by LC-MS. The ultraviolet (UV) detection was set at 260 nm, which corresponds to the absorption of the product 3-SPC. A prominent A_260 nm_ peak in the elution profile corresponding to an ESI (−) *m*/*z* ratio of 902.1 is consistent with the formation of 3-SPC in the full reaction mixture, but not in the negative controls omitting HpfD, HpfX, 3-HPS, CoA or NAD^+^ (Figures 2C, 2D, and S3).

### Activity assays for HpfYZ

**Figure 3:**
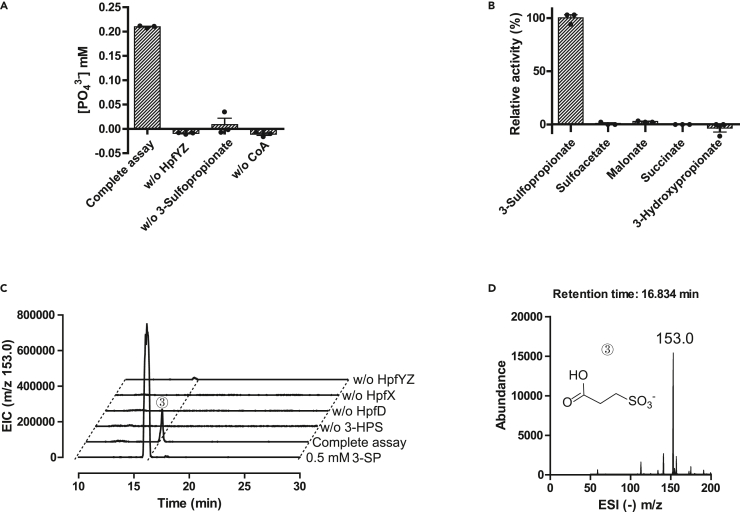
Activity assays of the ADP-forming 3-sulfopropionate-CoA ligase HpfYZ (A) Release of PO_4_^3−^ in the reaction of 3-SP, CoA and ATP catalyzed by HpfYZ, as detected using a phosphomolybdate colorimetric assay. The negative control omitting ATP was subtracted as the background value. (B) Substrate specificity for HpfYZ. Relative HpfYZ enzyme activities with different substrates. (C) HPLC elution profiles of the reaction of 3-HPS, CoA, ADP, NAD^+^ and phosphate catalyzed by HpfD, HpfX and HpfYZ, monitoring extracted ion counts of ESI negative mode. (D) Negative ionization mass spectra of peak 3 in c), corresponding to the reaction product 3-SP. The error bars represent standard error of mean.

AlphaFold3 structure predicts a HpfY/HpfZ heterodimeric assembly similar to other members of the succinate-CoA ligase family (Figure S4). The enzymatic activity of HpfYZ was assayed for its ability to catalyze the ATP-driven ligation of 3-SP and CoA, which is the reverse of its proposed physiological reaction. Incubation of purified HpfYZ with 3-SP, CoA, Mg^2+^ and ATP led to phosphate liberation, as detected by a phosphomolybdate colorimetric assay (Figure 3A), quantified using a standard curve (Figure S5). The negative control omitting ATP was subtracted as the background value. No reaction was observed in negative controls, in which either HpfYZ or one of the three substrates was omitted (Figure 3A).

Reaction kinetics was monitored using a pyruvate kinase and lactate dehydrogenase (PK-LDH) coupled spectrophotometric assay, in which the rate of NADH consumption reflects the rate of ADP production. The optimal reaction pH was determined to be 7.5 (Figure S6A). Activity was proportional to the amount of HpfYZ added (Figure S6B). The apparent Michaelis-Menten kinetic parameters of HpfYZ were measured with 2 μM HpfYZ, by varying the concentrations of one of the three substrates while maintaining the other two in excess (*k*_cat_ = 0.38 ± 0.01 s^−1^, *K*_M_ = 0.51 ± 0.07 mM for 3-SP; *k*_cat_ = 0.45 ± 0.04 s^−1^, *K*_M_ = 0.11 ± 0.03 mM for CoA; *k*_cat_ = 0.33 ± 0.01 s^−1^, *K*_M_ = 0.18 ± 0.14 mM for ATP) (Figures S6C–S6E). No activity was observed when 3-SP was replaced with sulfoacetate, succinate, malonate or 3-hydroxypropionate (Figure 3B), demonstrating that HpfYZ is highly specific for 3-SP.

### LC-MS assay for HpfD-HpfX-HpfYZ reaction

The substrate of HpfX, 3-SPA, was generated by HpfD-catalyzed oxidation of 3-HPS. The ability of the HpfX-HpfYZ system to convert 3-SPA into 3-SP was ascertained by incubating the purified enzymes (HpfD, HpfX, and HpfYZ) with 3-HPS, NAD^+^, CoA, Mg^2+^, phosphate and ADP, followed by analysis of the reaction product by LC-MS (Figure 3C). A prominent peak in the extracted ion chromatogram, co-eluting with the 3-SP standard and displaying an ESI (−) *m*/*z* ratio of 153.0 (Figures 3D and S7) is consistent with the formation of 3-SP in the full reaction mixture, but not in the negative controls.

### Activity assays for HpfYZ-HpfX reaction

HpfX was assayed for its ability to catalyze the reduction of 3-SPC, which is the reverse of its proposed physiological reaction. Incubation of purified HpfX and HpfYZ with Mg^2+^, K^+^, NADH, ATP, CoA and 3-SP led to the time-dependent decrease in absorbance at 340 nm (A_340 nm_), indicating reduction of 3-SPC accompanied by the consumption of NADH (Figure S8A). Substitution of NADH with NADPH significantly diminished the activity, demonstrating that HpfX is highly specific for NADH (Figure S8B). Overall, the biochemical assays demonstrate the ability of the HpfXYZ system to catalyze the reversible oxidation of 3-SPA to 3-SP, coupled to the reduction of NAD^+^ to NADH and phosphorylation of ADP to ATP.

### Analysis of the active site of HpfYZ

**Figure 4:**
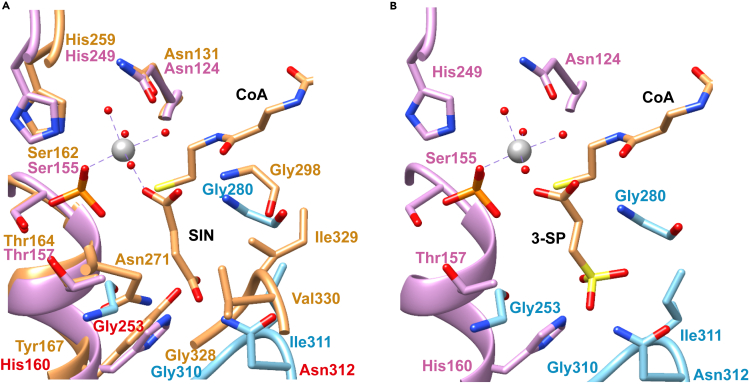
AlphaFold model of HpfYZ (A) Superimposition of the AlphaFold model of HpfYZ (cyan, HpfY; plum, HpfZ) with the crystal structure of succinyl-CoA synthetase (SCS) from *Sus scrofa* (tan, PDB: 5CAE). SIN (succinic acid), the substrate of SCS is labeled. And the magnesium ion is labeled in gray. Putative signature residues for HpfYZ are labeled in red. (B) Active site structure of HpfYZ. The SCS protein was hidden from a) and SIN was replaced with 3-SP *in situ*.

To gain insights into the substrate specificity of HpfYZ, its structural model was obtained from the AlphaFold database^33^ and overlaid with the crystal structure of *Sus scrofa* succinyl-CoA synthetase (SCS) in complex with succinate, phosphate, Mg^2+^ and CoA (PDB: 5CAE)^34^ (Figure 4A). Examination of the active site revealed the conservation of residues involved in binding of phosphate, Mg^2+^, and the substrate C1 carboxylate, which include S155, H249, N124 in HpfZ and G280 in HpfY, and are highly conserved in the SCS family (Pfam: PF08442, PF00549 and PF02629) (Figures 4, S9, and S10). To investigate the mode of binding of the carboxylate substrate, the putative 3-SP site of HpfYZ was estimated by alignment with the SCS structure (Figure 4B). In SCS, the succinate C4 carboxylate group interacts with the OH group of Y167, and with backbone amide groups of a GIV motif (Figures 4 and S10). Y167 is replaced with H160 in HpfZ, which may accommodate the bulkier sulfonate group of 3-SP, while the GIV motif is replaced with GIN (G310, I311, N312) in HpfY, with a possible interaction between the sulfonate group and the N312 side chain. Thus the residues H160 in HpfZ and N312 in HpfY may serve as a signature residues of HpfYZ to distinguish it from SCS.

### Growth of *E. gilvus* on SQ or on DHPS plus pyruvate

**Figure 5:**
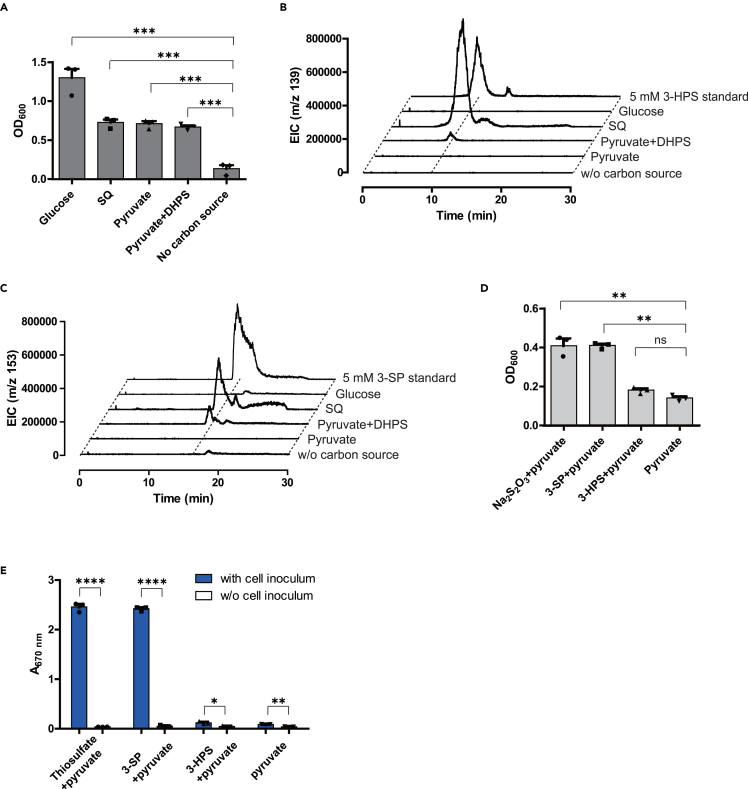
Growth of *E. gilvus* and *B. wadsworthia* (A) Growth of *E. gilvus* on different carbon and energy source. (B and C) HPLC elution profiles of the spent medium, monitoring the ion counts of ESI negative mode, showing the formation of 3-HPS in b) and 3-SP in c). (D) Growth of *B. wadsworthia* supported by different sulfur-containing STEAs. (E) Formation of H_2_S in the headspace gas accompanying reduction of various STEAs by *B. wadsworthia*, as detected by a methylene blue assay. Error bars reflect the SD values. Significance was determined by t test, ∗*p* < 0.1, ∗∗*p* < 0.01, ∗∗∗*p* < 0.001, and ∗∗∗∗*p* < 0.0001. Nonsignificant differences are indicated by ns.

Having determined the enzymatic activities of HpfXYZ and confirmed the enzymatic activity of HpfD, we next investigated the ability of *E. gilvus* to generate the predicted products 3-SP and 3-HPS. *E. gilvus* was grown in defined media containing either SQ or DHPS plus pyruvate as the carbon and energy source. The spent medium was analyzed by LC-MS, and concentration of the sulfonate products were estimated by comparing the peak area integration of samples relative to the standards. 3-SP and 3-HPS was detected for media containing either SQ (2 mM 3-SP, 9 mM 3-HPS) or DHPS (0.5 mM 3-SP, 1 mM 3-HPS), but not for negative controls containing either glucose or pyruvate as the sole carbon and energy source (Figures 5A, 5B, 5C, and S11).

### Growth of *B. wadsworthia* with 3-SP but not 3-HPS as a source of TEA

The conversion of SQ and DHPS to 3-SP and 3-HPS by anaerobic bacteria has implications regarding pathways for sulfonate sulfur mineralization in anaerobic environments. *B. wadsworthia* plays a prominent role in sulfonate sulfur mineralization in the human gut, and is able to utilize all four sulfoglycolytic products (DHPS, sulfolactate, isethionate, sulfoacetate) as sources of TEA, converting the sulfonate sulfur to H_2_S.^24^ We next investigated whether *B. wadsworthia* can also utilize 3-HPS and 3-SP as sources of TEA for anaerobic growth. Strikingly, no growth was observed with 3-HPS, while robust growth was observed with 3-SP (Figure 5D) accompanied detected of H_2_S in the headgas using a methylene blue colorimetric assay (Figure 5E).

### Presence of the bifurcated pathway for DHPS disproportionation in different bacteria

We next searched for the occurrence of gene clusters encoding the bifurcated DHPS degradation pathway in different bacteria. The genome neighborhood of 100 HpfG homologs belonging to the cluster UniRef 50_A0A318FL05 were examined using the Enzyme Function Initiative Genome Neighborhood Tool (EFI-GNT),^35^ and those containing homologs of HpfXYZ (Pfam: PF00171, PF08442 and/or PF02629) were collated (Figures S12 and 6; Table S1). Sequence alignments show that the proposed signature residues of HpfYZ (H160 in HpfZ and N312 in HpfY) are conserved. Interestingly, gene clusters containing HpfXYZ also invariably contain HpfD, suggesting that 3-SP is not produced in isolation but together with 3-HPS as part of a bifurcated pathway for DHPS disproportionation.

**Figure 6:**
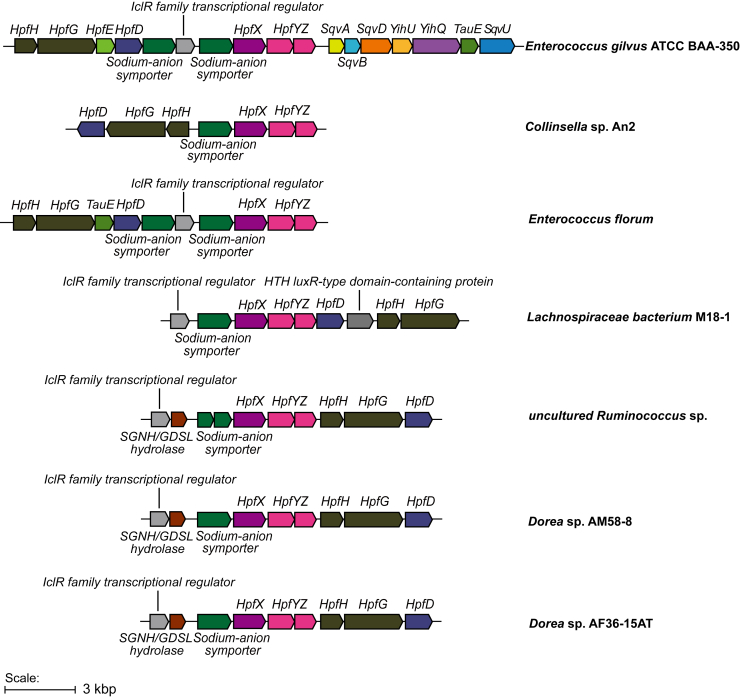
Genome neighborhoods of HpfY homologs Genome neighborhood analysis of HpfY associated with HpfX (Aldedh, Pfam: PF00171), HpfG (Gly_radical, Pfam: PF1228), HpfD (M_ADH, Pfam: PF00465), and SqvA (TAL_FSA, Pfam: PF00923).

## Discussion

Bacterial glycolytic degradation of SQ is thought to be a major source of C2 and C3 sulfonates in the environment.^4^^,^^5^ Our studies of the extended sulfoglycolytic pathway in *E. gilvus* adds 3-SP to the list of SQ-derived sulfonates, underscoring the complexity of organosulfur metabolism in bacterial consortia. In the *E. gilvus* pathway, SQ is first metabolized to DHPS via the sulfo-TAL pathway, followed by dehydration of DHPS to 3-SPA by HpfG,^27^ and a bifurcation in the pathway where 3-SPA is either reduced by HpfD to 3-HPS, or oxidized to by HpfXYZ to 3-SP, an SQ-derived sulfonate metabolite. Frommeyer et al. reported that SQ degradation by *E. gilvus* yields acetate and formate,^13^ consistent with a process where C1-3 of SQ is converted to acetate and formate via pyruvate formate lyase. Our experiments demonstrate that C4-6 of SQ is converted to DHPS, followed by disproportionation to 3-HPS and 3-SP, coupled to the formation of an additional 0.5 ATP. No sulfolactate was observed in growth experiments here, different from prior observation in *E. gilvus*.^13^ The bifurcated pathway thus theoretically allows these fermenting bacteria to balance NAD(H) levels while increasing ATP production. Analysis of the spent media shows that the amount of 3-HPS exceeds 3-SP, corresponding to a greater fraction of 3-SPA being reduced rather than oxidized. The excess reducing equivalents may originate from other components in the growth media (e.g., amino acids), though further investigation is required.

Apart from being a byproduct of bacterial SQ degradation, DHPS is also found as an osmolyte in diatoms,^36^^,^^37^ which are highly abundant primary producers in marine and freshwater bodies. DHPS degradation by aerobic bacteria involves either oxidation to sulfolactate followed by desulfonation by (2*R*)-sulfolactate sulfolyase (SuyAB),^38^^,^^39^ oxidation and decarboxylation to sulfoacetaldehyde followed by desulfonation by sulfoacetaldehyde acetyltransferase (Xsc), or conversion to cysteate and desulfonation by cysteate sulfolyase (CuyA).^39^ Meanwhile, DHPS degradation by the anaerobic SSRB involves either the sulfolactate pathway,^22^ or direct desulfonation catalyzed by the GRE (2*S*)-DHPS sulfolyase (HpsG).^27^ DHPS metabolism by fermenting bacteria was previously reported to involve dehydration to 3-SPA, followed by reduction to 3-HPS.^27^ Our experiments show that in *E. gilvus*, a fraction of 3-SPA is oxidized, resulting in the formation of both 3-HPS and 3-SP. Similar gene clusters for DHPS disproportionation are present in several gut bacteria from human (*Dorea* sp. AM58-8, *Dorea* sp. AF36-15AT, uncultured *Ruminococcus* sp.), mouse (*Lachnospiraceae bacterium* M18-1) and chicken (*Collinsella* sp. An2), and in *Enterococcus florum* (isolated from cotton flower) (Figure 6). HpfG-dependent extended sulfoglycolytic pathways for metabolism of SQ to 3-HPS are found in several bacteria including marine *Vibrio* and *Epulopiscium* sp.,^5^ while the variant for metabolism of SQ to 3-HPS and 3-SP is only present in *E. gilvus*.

The discovery that 3-HPS and 3-SP are products of anaerobic bacterial metabolism of the naturally abundant SQ and DHPS will motivate further studies into their roles in the sulfur cycle, particularly in anaerobic environments. 3-SP was previously identified as a byproduct generated when the non-natural C3 sulfonate homotaurine is metabolized as a nitrogen source by *Burkholderia* sp.^40^ and *Cupriavidus necator* H16.^41^ The pathway in *C. necator* involves an inducible homotaurine: 2-oxoglutarate aminotransferase and a non-acylating NAD(P)^+^-dependent 3-sulfopropionaldehyde dehydrogenase, homologs of enzymes involved in 4-aminobutyrate degradation.^41^ Although bacterial degradation of 3-HPS and 3-SP has not previously been reported, metabolism of homotaurine as a carbon source by the aerobic bacterium *Roseovarius nubinhibens* ISM was reported to involve 3-SP as an intermediate (Jutta Mayer, thesis dissertation, University of Konstanz), which is further degraded via sulfoacrylate and sulfolactate. Whether a similar pathway is involved in 3-SP dissimilation by *B. wadsworthia* and other SSRB remains to be investigated.

The HpfG-dependent formation of 3-HPS and 3-SP is expected to affect the cross-feeding of sulfonate substrates between fermenting bacteria and SSRB in the anaerobic human gut.^24^ Fermenting bacteria convert sulfonate-containing substrates such as SQ and taurine-conjugated bile salts to C3 and C2 sulfonates such as DHPS,^22^^,^^23^ taurine and isethionate,^42^^,^^43^^,^^44^ while SSRB such as *B. wadsworthia* catalyze desulfonation of these substrates and convert the sulfonate sulfur to H_2_S.^24^ Despite the ability of *B. wadsworthia* to degrade a wide range of sulfonates, it was unable to degrade 3-HPS. Thus the conversion of SQ and DHPS to 3-HPS is expected to render the sulfonate sulfur unavailable to *B. wadsworthia*, with impacts on H_2_S generation and proliferation of this pathobiont. SSRB have a widespread occurrence in the human intestinal microbiome, and despite their low abundance relative to fermenting bacteria, they play key roles in gut sulfur metabolism.^45^ Further studies are needed to determine the range of sulfonate substrates utilized by SSRBs, and the extent they are influenced by cross-feeding and competition from fermenting bacteria. Our findings contribute to the ongoing exploration of sulfur metabolism in the microbiome, opening new avenues for understanding and manipulating these complex biochemical pathways.

### Limitations of the study

We have attempted SDS-PAGE followed by proteomics analysis for showing if the enzymes of these bifurcated pathway are inducibly expressed upon growth on SQ, but have thus far been unable to obtain conclusive results, possibly due to the large number of enzymes involved in this bifurcated extended sulfoglycolysis pathway.

## Resource availability

### Lead contact

Further information and requests for resources and reagents should be directed to and will be fulfilled by the lead contact, Y.Z. (yan.zhang@tju.edu.cn).

### Materials availability

All of the reagents reported in this study are available from the lead or correspondence contact with Materials Transfer Agreement as long as stocks remain available.

### Data and code availability

• Source data underlying Figures 2, 3, and 5 have been deposited at Mendeley Data and are publicly available as of the date of publication. Accession link is listed in the key resources table.
• This paper does not report original code.
• Any additional information required to reanalyze the data reported in this paper is available from the lead contact upon request.

## Acknowledgments

We thank the instrument analytical center of the School of Pharmaceutical Science and Technology at Tianjin University for technical assistance. This work was supported by the 10.13039/501100012166National Key R&D Program of China 2020YFA0907900 (Y.Z), the 10.13039/501100001809National Natural Science Foundation of China (NSFC) Distinguished Young Scholar of China Program 32125002 (Y.Z), the National Research Foundation, Prime Minister’s Office, Singapore under its Campus for Research Excellence and Technological Enterprise (CREATE) program (CNSB) (Y.Z), the 10.13039/501100001809National Natural Science Foundation of China 32470026 (Y.T), the Tianjin University Independent Innovation Grant 2024XQM-0014 (Y.T), the Advanced Manufacturing and Engineering Programmatic Grant (A18A9b0060) (Y.W) and the funding support from 10.13039/501100001348Agency for Science, Technology and Research C211917011 (Y.W).

## Author contributions

Y.C., Y.W., and Y.Z. conceptualization; Y.C., T.Y., M.K., J.L., Y.Q., L.Z., R.C., H.M., and G.Q. data curation; Y.C., Y.W., R.C., and Y.T. formal analysis; Y.W., and Y.Z. funding acquisition; Y.C., Y.W., Y.T., and Y.Z. investigation; Y.C., Y.W., R.C., Y.T., and Y.Z. methodology; Y.Z. project administration; Y.W., Y.T., and Y.Z. resources; Y.W., Y.T., and Y.Z. supervision; Y.C., Y.W., Y.T., and Y.Z. validation; Y.C., Y.W., Y.T., and Y.Z. visualization; Y.C., Y.W., Y.T., and Y.Z. writing-original draft; Y.C., Y.W., F.L., Y.T., and Y.Z. writing review and editing.

## Declaration of interests

The authors declare that there are no conflicts of interest with the contents of this article.

## STAR★Methods

### Key resources table

**Table:** 

REAGENT or RESOURCE	SOURCE	IDENTIFIER
**Bacterial and virus strains**		

*Enterococcus gilvus*	DSMZ	DSM 15689
*Bacillus wadsworthia*	DSMZ	DSM 11045
*E. coli* DH5α	TransGen Biotech	Cat#CD501
*E. coli* BL21	Biomed	Cat#zc0380-m0243

**Chemicals, peptides, and recombinant proteins**		

Sulfoquinovose	ABCR	CAS: 3458-06-8
NADH sodium salt	MERYE	CAS: 76961-04-1
NAD^+^ sodium salt	Genview	CAS: 606-68-6
3-Hydroxypropionic acid	Adamas-beta	CAS: 503-66-2
Sulfoacetic acid	Sigma-Aldrich	CAS: 123-43-3
Malonic acid disodium salt	JIUDING	CAS: 141-95-7
3-Sulfopropanoic acid	BIDEpharm	CAS: 44826-45-1
Succinic acid	J&K Scientific	CAS: 110-15-6
Dihydroxypropanesulphonic acid	Enamine	CAS: 10296-76-1

**Deposited data**		

Source data obtained in this study	This paper	https://data.mendeley.com/datasets/jtd3khfznw/1

**Software and algorithms**		

Graphpad Prism 9	GraphPad	https://www.graphpad.com/
Adobe Illustrator 2021	Adobe Illustrator	https://www.adobe.com/cn/products/illustrator.html
Chimera 1.11.2	Chimera	https://www.cgl.ucsf.edu/chimera/
Chemdraw 2012	PerkinElmer	https://revvitysignals.com/products/research/chemdraw

### Experimental model and study participant details

#### Bacterial strains

Plasmids used in this study were synthesized and inserted into the *Ssp*I site of the modified pET28 vector HT by General Biosystems, Inc (Anhui, China). The proteins were expressed in *E.coli* BL21 (DE3) cells. *E*. *gilvus* (DSM 15689) and *B. wadsworthia* (DSM 11045) were used for the growth experiments.

### Method details

#### General

Lysogeny Broth (LB) was prepared with yeast extract and tryptone, sourced from Oxoid, England. The acetonitrile used for LC-MS analyses was procured as a high-purity solvent from Concord Technology (Minnesota, USA). The coenzymes NADH sodium salt and NAD^+^ sodium salt were obtained from Genview (Beijing, China) and MERYE (Shanghai, China), respectively. ADP was acquired from Solarbio (Beijing, China), ATP and CoA from Yuanye (Shanghai, China). The chemicals 3-HPS and 3-hydroxypropionic acid were sourced from Adamas-beta (Shanghai, China), 3-SP from BIDEpharm (Shanghai, China), sulfoacetic acid from Sigma-Aldrich (Shanghai, China), malonic acid disodium salt from JIUDING (Shanghai, China), succinic acid from J&K Scientific (Beijing, China), DHPS from Enamine (New Jersey, USA). Water used in this work was ultrapure deionized water from Millipore Direct-Q. Chromatographic purification of proteins was conducted using either an “ÄKTA pure” or “ÄKTA prime plus” FPLC system, equipped with appropriate columns from GE Healthcare (Illinois, USA). Protein concentrations were determined via absorbance at 280 nm, measured on an Ultramicro-UV-vis Spectrophotometer ND-100C from Miu Instruments (Hangzhou, China). The NADH-coupled enzymatic activity assays were performed by monitoring absorbance changes at 340 nm using a Tecan M200 plate reader.

#### Gene synthesis, molecular cloning and plasmid construction

DNA fragments encoding *E. coli* codon-optimized sequences of *E*. *gilvus* ATCC BAA-350. HpfD (*Eg*HpfD, Uniprot: R2XLL6), HpfX (*Eg*HpfX, Uniprot: R2XKF7) and HpfYZ (*Eg*HpfY, Uniprot: R2XLL0; *Eg*HpfZ, Uniprot: R2XZF8) were synthesized by General Biosystems (Anhui, China). These fragments were then cloned into the *Ssp*I site of the modified pET28 vector (HT plasmids) respectively, introducing an N-terminal His_6_-tag and a Tobacco Etch Virus (TEV) protease cleavage site before the target genes. HpfY and HpfZ were co-expressed from the same plasmid, incorporating an additional ribosome binding site (RBS, ATTAAAAAATAAGGAGGATTACCAT) between the genes of HpfY and HpfZ.

#### Protein expression and purification

A single colony of *E. coli* BL21 (DE3) transformant was inoculated into 5 mL LB medium containing 50 μg/mL kanamycin, and grown at 37°C for 4 h, then scaled up to 1 L of the fresh medium. The culture was incubated at 37°C with shaking at 220 rpm until the OD_600_ reached 0.6–0.8. The temperature was then decreased to 18°C, and isopropyl β-D-1-thiogalactopyranoside (IPTG) was added to a final concentration of 0.3 mM to trigger protein expression. After 16 h of induction, the cells were collected by centrifugation at 5,000 × *g* for 10 min at 4°C. The resulting cell pellet were resuspended in 10 mL of lysis buffer [50 mM Tris-HCl, pH 8.0, 200 mM KCl, 1 mM phenylmethanesulfonyl fluoride, 0.2 mg/mL lysozyme, 0.03% Triton X-100, and 0.02 mg/mL of DNase I], and frozen in a −80°C freezer.

For HpfD and HpfX purification, the frozen cell suspension was thawed and subjected to lysis by incubation at room temperature (RT) for 50 min. To precipitate and remove nucleic acids, 1% streptomycin sulfate was added, followed by centrifugation at 12,000 × *g* for 15 min at 4°C. The clarified supernatant was filtered through a 0.45 μm filter before being loaded onto a pre-equilibrated 5 mL TALON Co^2+^ column (Clontech Laboratories, Inc.) using buffer A [20 mM Tris-HCl, pH 7.5, and 0.2 M KCl]. The column was washed with 10 column volumes (CV) of buffer A, and protein was subsequently eluted using 5 CV of buffer B [20 mM Tris-HCl, pH 7.5, 5 mM BME, 0.2 M KCl and 150 mM imidazole]. For HpfYZ purification, the supernatant was applied to a 5 mL Ni-NTA column, washed with 5 CV buffer A plus 100 mM imidazole and then eluted with 5 CV buffer A containing 250 mM imidazole. The eluates were collected and dialyzed against 2 L of buffer A supplemented with 5 mM BME at 4°C for 3 h, then concentrated and flash-frozen in aliquots in liquid nitrogen, and stored at −80°C.

The purified proteins were analyzed by SDS-PAGE analysis using a commercial gel (YoungPAGE, Bis-Tris, 4–20%). Their concentrations were determined based on absorptions at 280 nm, measured using an Ultramicro-UV-Vis Spectrophotometer ND-100C. Their extinction coefficients were calculated utilizing the ProtParam tool available on the ExPASy website (http://www.expasy.org/protparam/) [HpfD (ε_280 nm_ = 27,850 M^−1^cm^−1^), HpfX (ε_280 nm_ = 31,400 M^−1^cm^−1^), HpfYZ (ε_280 nm_ = 30,830 M^−1^cm^−1^)].

#### Activity assays for HpfD

In a typical assay setup, a 200 μL reaction mixture was prepared, which contained 50 mM 3-(cyclohexylamino)-2-hydroxy-1-propanesulfonic acid (CAPSO) at pH 9.5, 4 mM NAD^+^, and 40 mM 3-HPS. The reaction commenced with the introduction of 0.1 μM HpfD, and the absorbance at 340 nm was continuously monitored for 2 min at RT using a Tecan M200 plate reader. To ascertain the optimal pH for HpfD activity, assays were conducted with 0.1 μM HpfD across various buffers at 50 mM concentration: Tris-HCl, pH 8.0; CAPSO, pH 8.5, 9.0 and 9.5; 3-(cyclohexylamino)-1-propyl sulfonic acid (CAPS), pH 10.0 and 10.5. Enzyme dose-dependent assays were carried out by varying the enzyme concentrations from 0.025 to 0.1 μM. To obtain the apparent Michaelis-Menten kinetic constants, the concentration of one substrate was altered while maintaining a saturating concentration of the other substrate. The 3-HPS concentration was varied from 0 to 100 mM in the presence of 4 mM of NAD^+^. The concentration of NAD^+^ was adjusted from 0 to 8 mM in the presence of 40 mM 3-HPS. The ΔA_340 nm_ and the extinction coefficient of NADH (ε_340 nm_ = 6,220 M^−1^cm^−1^) were used to calculate the reaction rates.

#### LC-MS assay for HpfD

A 50 μL reaction mixture comprising 50 mM CAPSO, pH 9.5, 2 μM HpfD, 50 mM 3-HPS, 4 mM NAD^+^ was incubated at RT for 30 min. For negative controls, HpfD, NAD^+^ or 3-HPS was individually excluded from the reaction mixture. The reaction solution was mixed with 550 μL 0.73 M sodium acetate buffer at pH 5.0 and 400 μL freshly prepared 2,4-dinitrophenylhydrazine (DNPH) solution (10 mg DNPH dissolved in 25 mL methanol). The mixture was incubated in water bath at 50°C for 1 h and then filtered through a 0.22 μm nylon membrane filter before LC-MS analysis, which was performed on an Agilent 6420 triple quadrupole LC-MS instrument (Agilent Technologies). The drying gas temperature was maintained at 330°C with a flow rate of 10 L/min and a nebulizer pressure of 45 psi. Chromatographic separation was achieved using an Agilent ZORBAX SB-C18 column (4.6 × 250 mm), employing a linear gradient from 15% to 80% solvent B over 20 min, where solvent A was 0.1% formic acid in water and solvent B was acetonitrile, at a flow rate of 1 mL/min. UV detection was set at 360 nm, and the mass spectrometer operated in the ESI negative mode.

#### LC-MS assay for the HpfD-HpfX coupling reaction

A 200 μL reaction mixture containing 50 mM CAPSO, pH 9.5, 40 mM 3-HPS, 0.5 mM CoA, 4 mM NAD^+^, 1 μM each of HpfD and HpfX was incubated at RT for 20 min. For negative controls, HpfD, HpfX, 3-HPS, CoA or NAD^+^ was individually excluded from the mixtures. Protein was removed by extracting with an equal volume of phenol/chloroform/isopentanol (25:24:1). After centrifugation, the aqueous layer was passed through a 0.22 μm nylon membrane filter in preparation for LC-MS analysis conducted on an Agilent 6420 triple quadrupole LC-MS instrument. The drying gas temperature was set to 330°C, with a flow rate of 10 L/min and a nebulizer pressure of 45 psi. Chromatographic separation was achieved using an Agilent ZORBAX SB-C18 column (4.6 × 250 mm), applying a linear gradient from 2% to 15% solvent B over 20 min, where solvent A was 10 mM ammonium acetate in water and solvent B was acetonitrile, at a flow rate of 0.75 mL/min. UV detection was set at 260 nm, and the mass spectrometer operated in the ESI negative mode. The MS2 data of the product 3-SPC was obtained by using a Q Exactive HF/UltiMate 3000 RSLCnano (Thermo Fisher) instrument in the ESI positive mode.

#### A colorimetric phosphomolybdate assay for HpfYZ

To establish the standard curve of PO_4_^3−^, a molybdic acid colorimetric solution was freshly prepared. Typically, 1 mL solution A, comprising 10% ammonium molybdate solution in 10 N H_2_SO_4_, was mixed into 8 mL deionized water. Subsequently, 0.5 g iron (II) sulfate heptahydrate (FeSO_4_·7H_2_O) was dissolved, and the total volume was adjusted to 10 mL with deionized water. Various concentrations of Na_3_PO_4_ ranging from 0 to 2 mM were mixed with the colorimetric solution in a 1:1 volume ratio. The resulting mixture was incubated at 25°C for 10 min, after which 200 μL of the mixture was transferred to a 96 well plate and the absorbance at 660 nm was measured. The baseline absorbance measured at 0 μM PO_4_^3−^ was subtracted to obtain ΔA_660 nm_ for each PO_4_^3−^ concentration. For the HpfYZ assay, a 105 μL reaction mixture containing 50 mM Tris-HCl, pH 7.5, 2 μM HpfYZ, 1 mM 3-SP, 0.8 mM CoA, 1 mM ATP, 10 mM MgCl_2_ and 50 mM KCl was incubated at RT for 20 min 105 μL freshly prepared molybdate colorimetric solution was added to stop the reaction. After incubation at 25°C for 10 min, 200 μL of the mixture was measured for A_660 nm_ in a 96 well plate with a Tecan M200 plate reader. The A_660 nm_ for the sample without ATP was subtracted to obtain ΔA_660 nm_, which was used to calculate the PO_4_^3−^production in the enzyme reaction referring to the standard curve.

#### Activity assays for HpfYZ

In a typical assay, a 200 μL reaction mixture containing 50 mM Tris-HCl, pH 7.5, 0.4 mM NADH, 0.4 mM CoA, 2 mM 3-SP, 2 mM ATP, 2 mM phosphoenolpyruvate (PEP), 10 mM MgCl_2_, 50 mM KCl, and 5 units of pyruvate kinase/lactate dehydrogenase enzymes (PK-LDH, Sigma-Aldrich) was prepared. The reaction was initiated by addition of 2 μM HpfYZ, and the absorbance at 340 nm was monitored using a Tecan M200 plate reader over 2 min at RT. The optimal pH was determined by the HpfYZ activity assays using 50 mM concentrations of various buffers: MOPS, pH 6.0 and 6.5; Tris-HCl, pH 7.0, 7.5, 8.0 and 8.5; CAPSO, pH 9.5. Enzyme dose dependent assays were carried out similarly varying the enzyme concentrations in the range from 0.5 to 4 μM. The substrate specificity assays were performed with various substrate analogs including 3-SP, sulfoacetate, succinate, malonate and 3-hydroxypropionate. To obtain the Michaelis-Menten kinetic constants, 3-SP, ATP, and CoA were varied in the ranges of 0 to 8, 0 to 4, and 0 to 0.6 mM respectively, with the other two substrates in saturation (2 mM 3-SP, 2 mM ATP and 0.4 mM CoA). ΔA_340nm_ and the extinction coefficient of NADH were used to calculate the rates of the reactions.

#### LC-MS assay for the HpfD-HpfX-HpfYZ coupling reaction

A 100 μL reaction mixture containing 50 mM CAPSO, pH 9.5, 1 μM HpfD, 1 μM HpfX, 40 mM 3-HPS, 0.8 mM CoA, 2 mM NAD^+^ was incubated at RT for 20 min, to which 100 μL of 100 mM Na_2_HPO_4_, pH 7.5, 10 mM MgCl_2_, 50 mM KCl, 2 mM ADP, and 1 μM HpfYZ was added, mixed and incubated for another 20 min at RT. One of the three enzymes, NAD^+^, or 3-HPS was omitted in the negative controls. Protein was precipitated using 200 μL acetonitrile and removed by centrifugation. The supernatant was filtered through a 0.22 μm nylon membrane filter prior to LC-MS analysis with a ZIC-HILIC column (5 mm, 200 Å, 150 × 4.6 mm; Merck) on an Agilent 6420 Triple Quadrupole LC-MS instrument. The drying gas temperature was maintained at 300°C with a flow rate of 9 L/min and a nebulizer pressure of 15 psi. The HPLC was programmed to adjust solvent B from 90% to 70% in 10 min and then from 70% to 50% in 20 min. Solvent A consisted of 90% 20 mM ammonium acetate and 10% acetonitrile, and solvent B was acetonitrile, with a flow rate maintained at 0.5 mL/min. The mass spectrometer was run in the ESI negative mode. The MS2 data of the product 3-SP was obtained by using a Q Exactive HF/UltiMate 3000 RSLCnano (Thermo Fisher) instrument.

#### Colorimetric assay for the HpfYZ-HpfX coupling reverse reaction

In a typical assay, a 200 μL reaction mixture consisting of 50 mM Tris, pH 7.5, 10 mM MgCl_2_, 50 mM KCl, 2 mM ATP, 0.4 mM CoA, 0.4 mM NADH and 10 mM 3-SP was prepared. The enzymatic reaction was initiated by introducing 5 μM each of HpfYZ and HpfX, and the absorbance at 340 nm was continuously monitored for a duration of 2 min at RT using a Tecan M200 plate reader. One of the enzymes or substrates was omitted as a negative control.

#### Growth of *B. wadsworthia* with different TEAs

Empty bottles (10 mL) used for anaerobic cultures were taken into a nitrogen glovebox 3 days before use. These bottles were then securely sealed, taken out of the glove box, and sterilized via autoclaving. Both ABB medium and DSM 503 medium, excluding components sensitive to heat, were prepared in 500 mL sealed bottles in the glovebox and autoclaved. Heat-sensitive components were dissolved in degasified H_2_O and filter-sterilized in the glove box. *B. wadsworthia* RZATAU (DSM 11045) cells were initially inoculated into the ABB medium contained in the 10 mL bottle and cultivated anaerobically at 37°C for 3 to 5 days. Then, 100 μL portions of the starter culture were transferred into four anaerobic bottles each containing 5 mL modified DSM 503 medium supplemented with 60 mM Na-formate, 200 μg/L 1,4-naphthochinone and 20 mM sodium pyruvate as the electron donor and carbon source. To test the source of TEA, 20 mM Na_2_S_2_O_3_, 3-SP, and 3-HPS were each added to one of the four bottles and the remaining bottle of culture without TEA was used as a negative control. The OD_600_ of each culture was recorded 4 days after and a photograph of the cultures was taken before the methylene blue assay for H_2_S in the headspace gas.^46^^,^^47^

#### Growth of *E*. *gilvus* with different carbon sources

*E. gilvus* strain (DSM 15689) was purchased from DSMZ. The rich medium (DSM 92 medium) was prepared by dissolving 3 g trypticase soy broth and 0.3 g yeast extract in 100 mL distilled water and autoclaving. The simplified defined medium (SDM), which includes amino acids, vitamins, nucleic acid bases, minerals, and 20 mM glucose^48^ was prepared in the glovebox and filter-sterilized. *E. gilvus* cells were inoculated into 5 mL rich medium and grown overnight in a 37°C incubator. Then 20-μL portions of the culture were transferred into 5 mL of SDM omitting glucose, SDM, SDM with 20 mM SQ, pyruvate, or pyruvate plus DHPS in substitution of glucose as the carbon source. The OD_600_ of each culture was recorded 4 days after.

#### Bioinformatics

*Eg*HpfY (Uniprot: R2XLL0) sequence was blasted using the network-based Enzyme Function Initiative-Enzyme Similarity Tool (EFI-EST). Sequence similarity networks (SSNs) of a total of 8,120 sequences were constructed using EFI-EST tool. The choice of an E-value cut-off of 10^−80^ in this case resulted in grouping sequences with a similarity greater than 50% into a cluster. The 80% similarity representative node (RepNode) network was displayed using Cytoscape v3.519. HpfY was labeled based on the *Eg*HpfY sequence. The information regarding the genome neighborhood within a 10 open reading frame (10-ORF) window was inserted using EFI-GNT.

### Quantification and statistical analysis

The data analysis was performed by using GraphPad Prism 9 software. The biochemistry experiments were repeated at least twice and the data were expressed as the mean ± SEM. Bar graphs and scatter blots represent the mean ± standard deviation (SD), when indicated. ANOVA was used followed by Tukey’s multiple comparison post-test comparing all pairs of conditions when multiple conditions were compared. Significant differences were indicated according to the *p*-values by asterisks with ∗∗∗∗ for *p* < 0.0001, ∗∗∗ for 0.0001 < *p* < 0.001, ∗∗ for 0.001 < *p* < 0.01, and ∗ for 0.01 < *p* < 0.05. Non-significant differences are indicated by n.s.
