# Supplementary material for: Study of sulfoglycolysis in Enterococcus gilvus reveals a widespread bifurcated pathway for dihydroxypropanesulfonate degradation
Source: iScience. 2024 Sep 21;27(10):111010. doi: 10.1016/j.isci.2024.111010 (PMC11489063; doi:10.1016/j.isci.2024.111010)
Supplement: Document S1. Figures S1–S12, Tables S1, and S2 [file mmc1.pdf]

iScience, Volume *27*

Supplemental information

Study of sulfoglycolysis in *Enterococcus gilvus* reveals a widespread bifurcated pathway for dihydroxypropanesulfonate degradation

Yiwei Chen, Ruoxing Chu, Kailiang Ma, Li Jiang, Qiaoyu Yang, Zhi Li, Min Hu, Qiuyi Guo, Fengxia Lu, Yifeng Wei, Yan Zhang, and Yang Tong

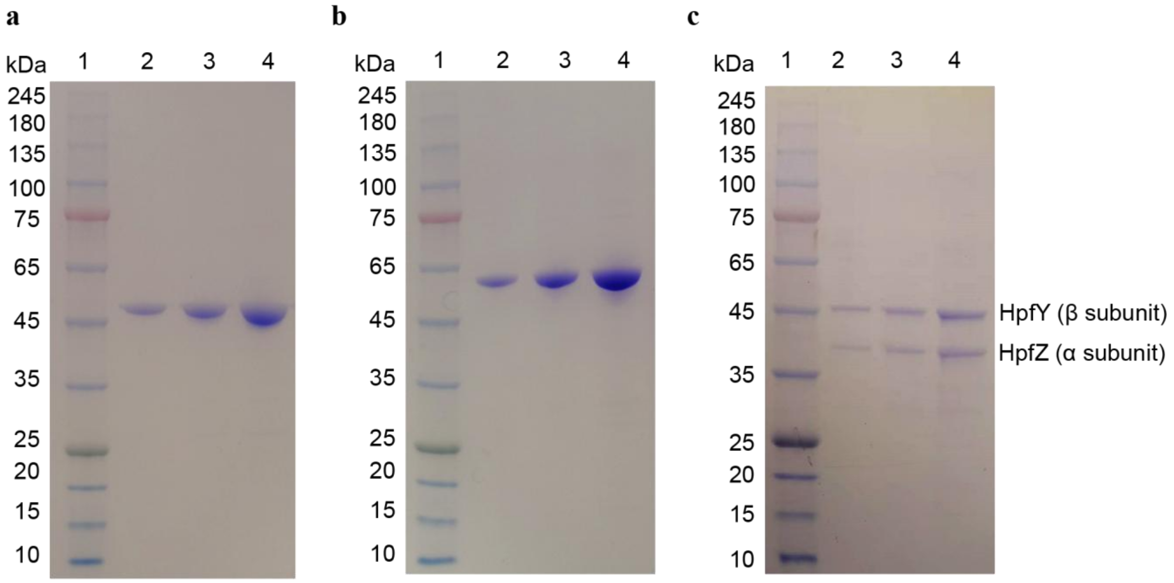

Figure S1. **SDS-PAGE analyses of purified HpfD**, **HpfX and HpfYZ (Related to Figure 1, Figure2, and Figure 3). a)** HpfD. **b)** HpfX. **c)** HpfYZ. A 4-20% gradient gel (Bis-Tris) with: lane 1, protein molecular weight marker; and lane 2-4: 1, 2, 4 μg of the respective protein of interest.

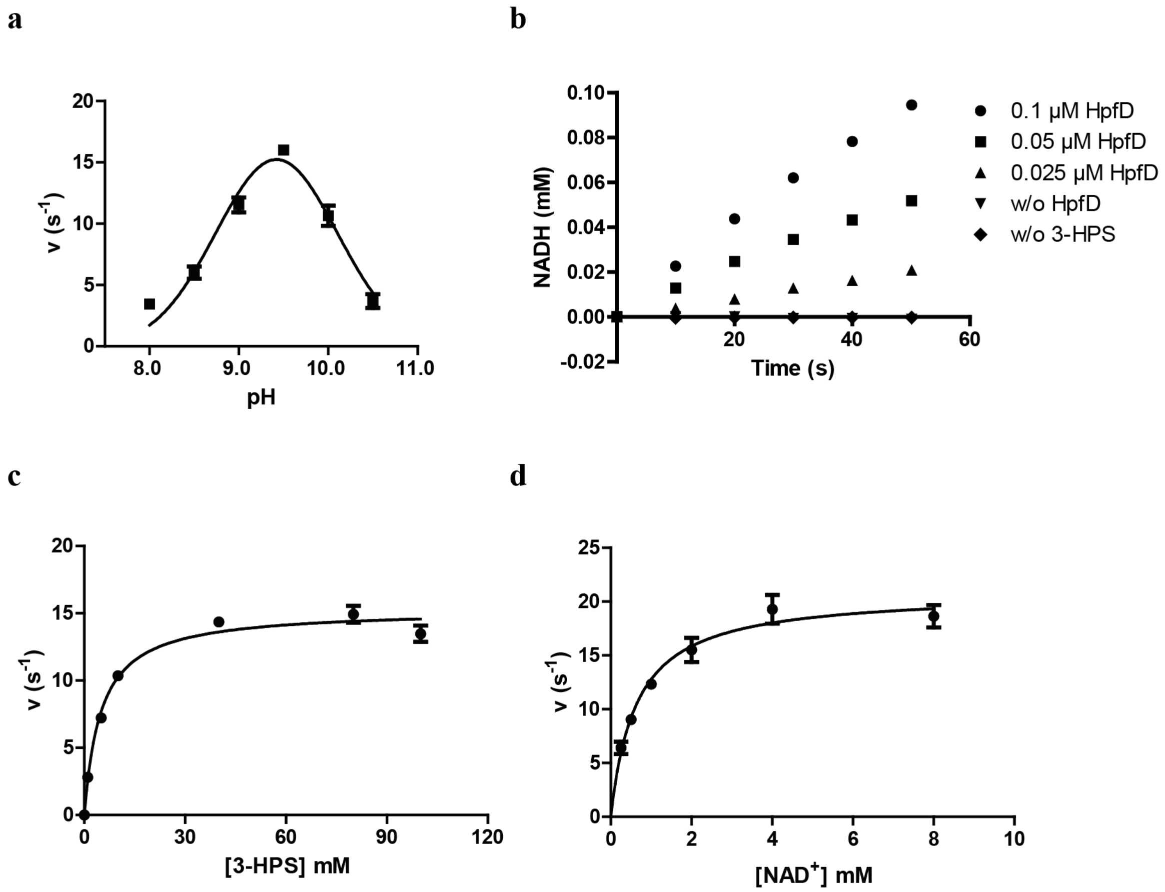

Figure S2. **Activity assays of HpfD (Related to Figure 2). a)** Effect of pH on HpfD 3-HPS dehydrogenase activity. The increase in A_{340} was recorded and used to calculate the activity of HpfD under different pH conditions. **b)** Enzyme dose-dependence of the oxidative reaction catalyzed by HpfD. **c-d)** Michaelis-Menten kinetics of HpfD with substrate 3-HPS and NAD^+. The reaction was monitored by measuring the increase of absorbance at 340 nm corresponding to NADH formation. Data are represented as mean ± SEM.

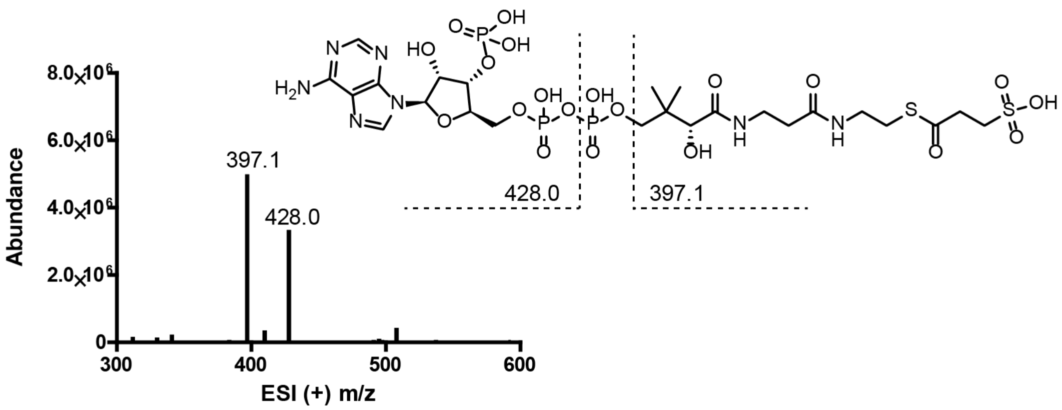

Figure S3. **The MS2 data of 3-sulfopropionyl-CoA (Related to Figure 2).**

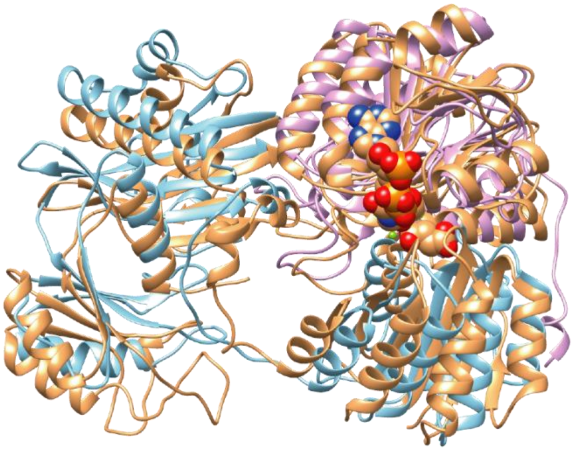

Figure S4. **Biomolecular assembly of HpfYZ (Related to Figure 4).** Superposition of the AlphaFold3 structure of HpfYZ (cyan, HpfY; plum, HpfZ) with succinyl-CoA synthetase (subunit alpha and beta) from *Sus scrofa* (tan, PDB: 5CAE). The bound CoA and succinic acid are shown as spheres.

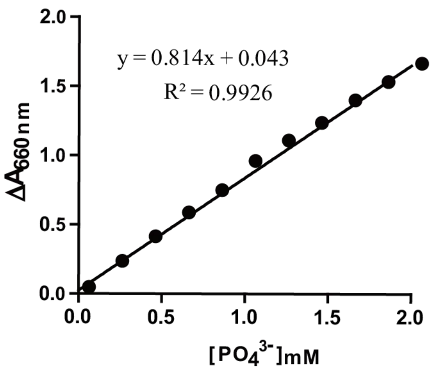

Figure S5. **Standard curve of PO_4^{3-} in the phosphomolybdate colorimetric assay (Related to Figure 3).** A range of concentrations (0-2.0 mM) of K_3PO_4 was used.

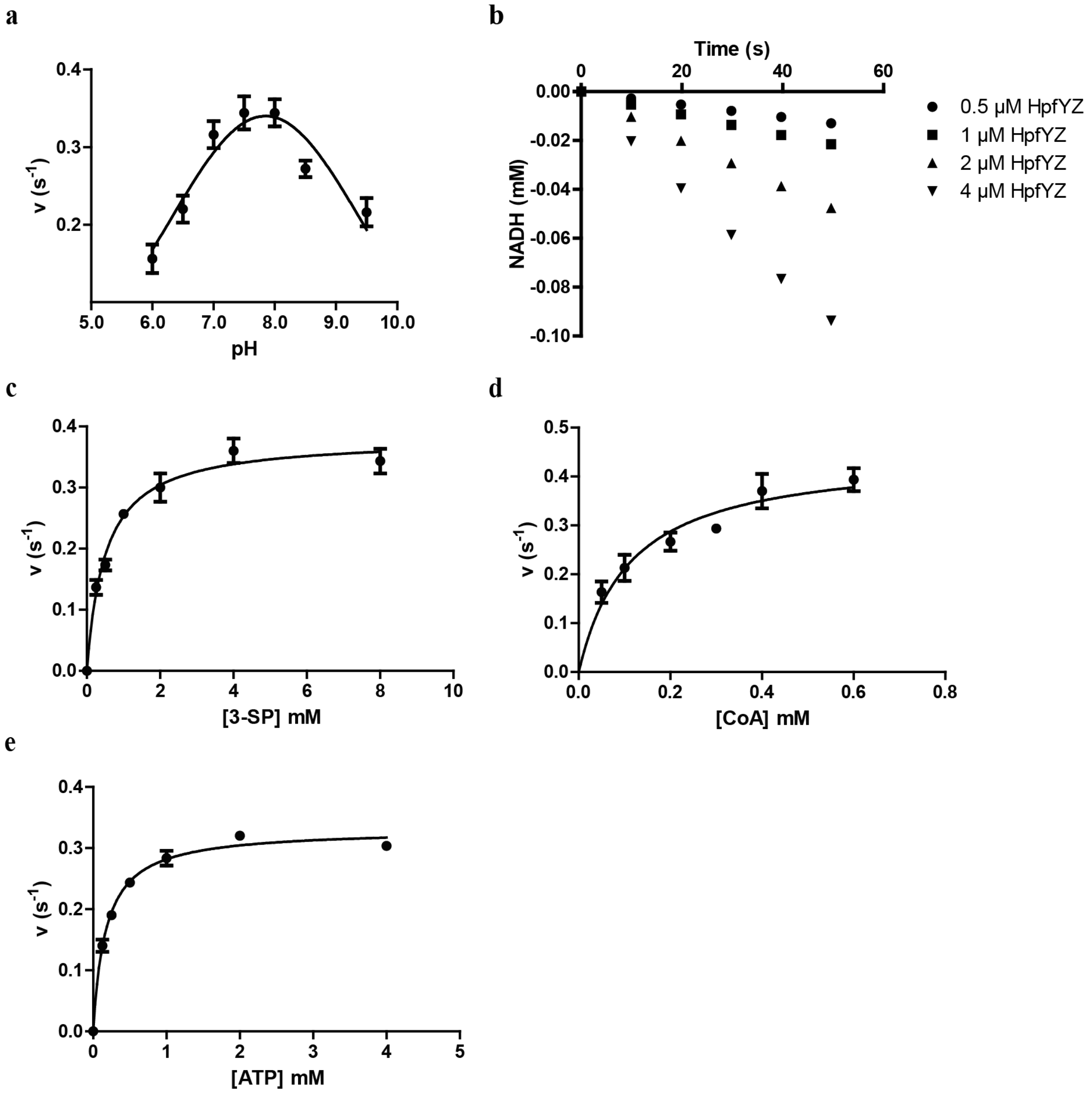

Figure S6. **Michaelis-Menten kinetics and pH dependent of HpfYZ (Related to Figure 3).** PK-LDH coupled activity assay was used for detection of ADP generated by HpfYZ-catalyzed 3-SP. The decrease of A_{340} was recorded and used to calculate the activity of HpfYZ under different pH conditions. **a)** Effects of pH on HpfYZ activity of 3-sulfopropionate-CoA ligase activity. **b)** Enzyme dose-dependence of the ligase reaction catalyzed by HpfYZ. **c)** Kinetic assays varying the concentration of 3-SP with a fixed concentration of 2 mM ATP and 0.4 mM CoA. **d)** Kinetic assays varying the concentration of CoA with a fixed concentration of 2 mM 3-SP and 2 mM ATP. **e)** Kinetic assays varying the concentration of ATP with a fixed concentration of 2 mM 3-SP and 0.4 mM CoA. All assays were carried out in triplicate. The error bars represent standard error of mean.

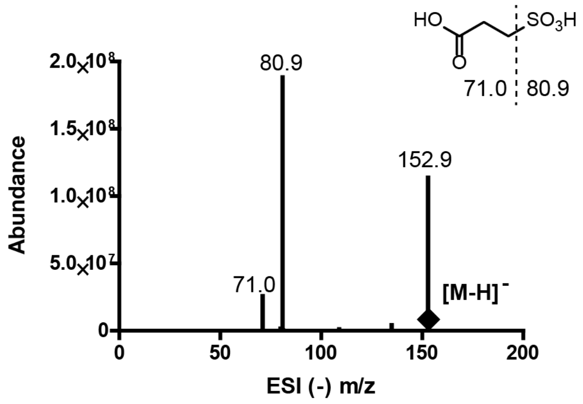

Figure S7. **The MS2 data of 3-sulfopropanoic acid (Related to Figure 3).**

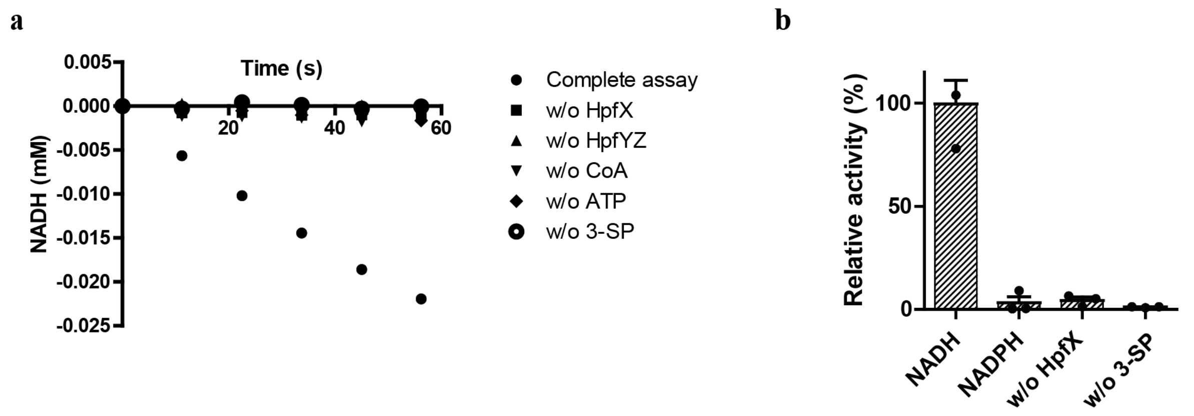

Figure S8. **Activity assays of HpfYZ-HpfX coupling reaction (Related to Figure 3). a)** Activity assays of HpfYZ-HpfX coupling reaction, assayed by monitoring the absorbance at 340 nm of the decrease of NADH. **b)** Reaction requirements of HpfX: complete assay mixture and controls omitting enzyme or substrate, or replacing NADH with NADPH. These assays were carried out in triplicate. Data are represented as mean ± SEM.

```
SqwK          F--DGDIGLMCAGAGLTTTVYDLIHYEGGTVANYLEFGGPNY-KKAVKAMEICLKVP-SK    297
P53590        L--DGNIACFVNGAGLAMATCDIIFLNGGKPANFLDLGGGVKESQVYQAFKLLTADPKVE    357
HpfY          VDPKGNIGTMAGGAGIGMATMDTVFHYGGRVNNFLDLGGGVTSEKTYQAMRILLENENTD    301
A0A4P5P8Q1    VDPEGDIGTMAGGAGIGMATMDTIFHYGGRVNNFLDLGGGVTAEKTYQAMRILLQNERTS    301
R9JZX3        LDPEGDIGTMAGGAGIGMATMDTIRHYGGRVNNFLDLGGGVTAEKTYQAMRILLQNKKTD    301
F0Z279        LNPEGDIGTMAGGAGIGMATMDTIRHYGGCVNNFLDLGGGVTAEKTYHAMKILLQNKSTN    301
              .  .*:*. :  ***:  :. * :   **   *:*::**    .:. :*:.:       .

SqwK          VILIVTFGTIARADVMAEGIVEAIKKLNPDRPIVTCIRGTNEAHAVELLKEAGLTP--LF    355
P53590        AILVNIFGGIVNCAIIANGITKACRELELKVPLVVRLEGTNVHEAQNILTNSGLPITSAV    417
HpfY          YILINIFGGINNCADMAEGITRAYKESGISKTVVVKSRGFNQEQGWSMYKELGFPQAKYG    361
A0A4P5P8Q1    YILVNIFGGINNCADMAEGITRAYKELGIPKTVVIKSRGFNQEQGWDMYQALGFPQTKYG    361
R9JZX3        YILVNVFGGINNCAEMAEGISRAYKEVGSSKPVAVKSRGFNQEQGWAIYEELGFAQTKFG    361
F0Z279        YILVNVFGGINNCADMAEGIARAYKELGIQKTVVVKSRGFNQEQGWTIYENLGFLQTKYG    361
               **:  ** * ..  :*:** .* ::      :.   .* *  ..  :    *:
```

Figure S9. **Multiple sequence alignments of putative HpfYs, SqwK and a succinyl-CoA ligase beta subunit (Related to Figure 4).** SqwK, the sulfoacetate-CoA ligase beta subunits from *Acholeplasma* sp. P53590, the succinyl-CoA synthetase beta subunit from *Sus scrofa* (PDB: 5CAE). Putative 3-sulfopropionate-CoA ligase beta subunits A0A4P5P8Q1, R9JZX3, F0Z279 from *Enterococcus florum*, *Lachnospiraceae bacterium* M18-1 and *Clostridium* sp. D5 respectively. A glycine residue with its backbone amide coordinating the carboxyl group of the substrate is highly conserved in all enzymes and highlighted in yellow. Key residues coordinating the sulfonic group of 3-SP in HpfYs are labeled in red.

```
O19069      VPPPFAAAAINEAIDAEVPLVVCITEGIPQQDMVRVKHRLLRQGKTRLIGPNCPGVINPG    179
SqwL        VPPKMTKDAVFEALEAGIKKIVTIADGIPLHEMMEIRQRALEEN-AFVVGGNTSGVISPK    131
R9JZ61      VPKQFVHDAAIAALDEGIRLLVIITEFVPVMDALHIVNHAKQTG-ARVVGPNTIGVISPG    132
F0Z280      VPPKFVKDAAMRALHEGIKLLVIITEFVPVLDVLEIVNEAKILG-AKVVGPNTIGIISPG    121
HpfZ        TPPRFVKDAAIQALQSGIELLVIITEFVPVLDTLEIVTKAKKLG-AKVVGPNTIGVIAPG    132
A0A4V0WPL1  TPPQFVKEAAIQALQAGIQLLVVITEFVPVMDTLHIVHEANRAG-ARIVGPNTIGIISPG    132
            .*  :.  *   *:.  :  :* *:: :*  : :.:  .    . : ::* *  *:* *

O19069      ECKIGIMPGH---IHKKGRIGIVSRSGTLTYEAVHQTTQVGLGQSLCVGIGGDPFNGTDF    236
SqwL        EAMMGSFPHWIERVYKKGSIGVMTRSGSLTNEVTAMIVEAGYGVSSLIGVGGDPVPGARF    191
R9JZ61      KAKIGIMPDY---IYGQGHIGIISRSGTLTHETASNLTFKGYGLSTCVGIGGDSIIGMNH    189
F0Z280      KSKLGIMPDY---IYGKGHIGIISRSGTLTHETASNLTFKGFGLSTCVGIGGDSIIGMNH    178
HpfZ        KSKLGIMPAD---IYGKGRIGIISRSGTLTHETASNLTFKGYGLSTCVGIGGDSIVGMDH    189
A0A4V0WPL1  KSKLGIMPAD---IYGKGHIGIISRSGTLTHETASNLTFKGYGLSTCVGIGGDSIVGMNH    189
            :. :* :*     :: :* **:::***:** *..   .  * * *  :*:*** . *  .

O19069      MGHAGAIIAGGKGGAKEKITALQSAGVVVSMSPAQLGTTIYKEFEKR----KML------    346
SqwL        MGHAGAIITGGKGSVQNKIEMLEKAGAKVADRPRKVGKLLEELGVTKD-----------    296
R9JZ61      MGHAGAIVSGGMGTVKSKVAALEEAGVTVCPTLGKIVENIAEYNMRTNGRLQTLKPQID-    305
F0Z280      MGHAGAIVSGGMGTVKSKVAALEDAGVTVCPTLGKIVECIDEYNIHTDGRLKTLAPQID-    294
HpfZ        MGHAGAIVSGNMGTVKSKVEALEDAGVTVCPTLGKIVEFMEKTNSQTNGKLRSLEPKVDA    306
A0A4V0WPL1  MGHAGAIVSGDMGTVKSKVAALEDAGVVVCPTLGKIVEFMDEHNGQLGGRLKTLEPREDE    306
            *******::*. * .:.*:  *:.**. *.    ::   : :
```

Figure S10. **Multiple sequence alignments of putative HpfZs, SqwL and a succinyl-CoA ligase alpha subunit (Related to Figure 4).** SqwL, the sulfoacetate-CoA ligase alpha subunits from *Acholeplasma* sp. O19069, the succinyl-CoA synthetase alpha subunit from *Sus scrofa* (PDB: 5CAE). Putative 3-sulfopropionate-CoA ligase alpha subunits A0A4V0WPL1, R9JZ61, F0Z280 from *Enterococcus florum*, *Lachnospiraceae bacterium* M18-1 and *Clostridium* sp. D5 respectively. Conserved residues that coordinate phosphate or Mg^{2+} are highlighted in yellow. Key residues interacting with the 3-SP sulfonic group are labeled in red.

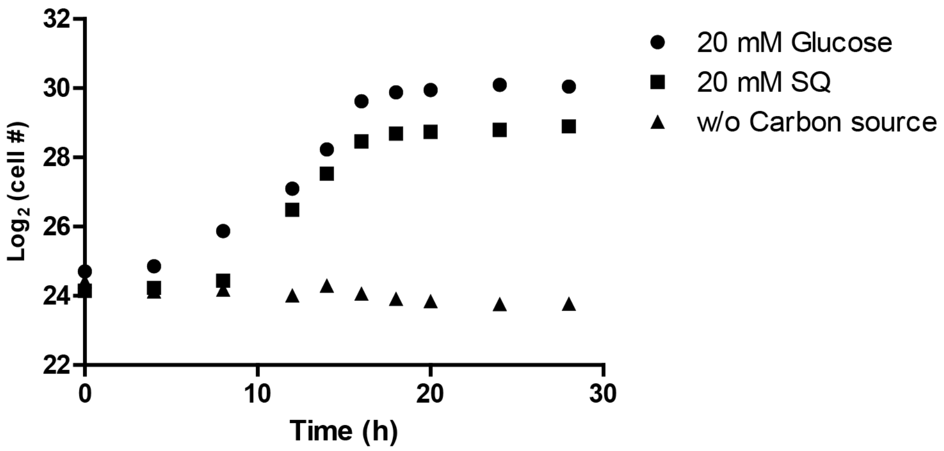

Figure S11. **Growth curves of *E. gilvus* in defined media with various carbon sources (Related to Figure 5).**

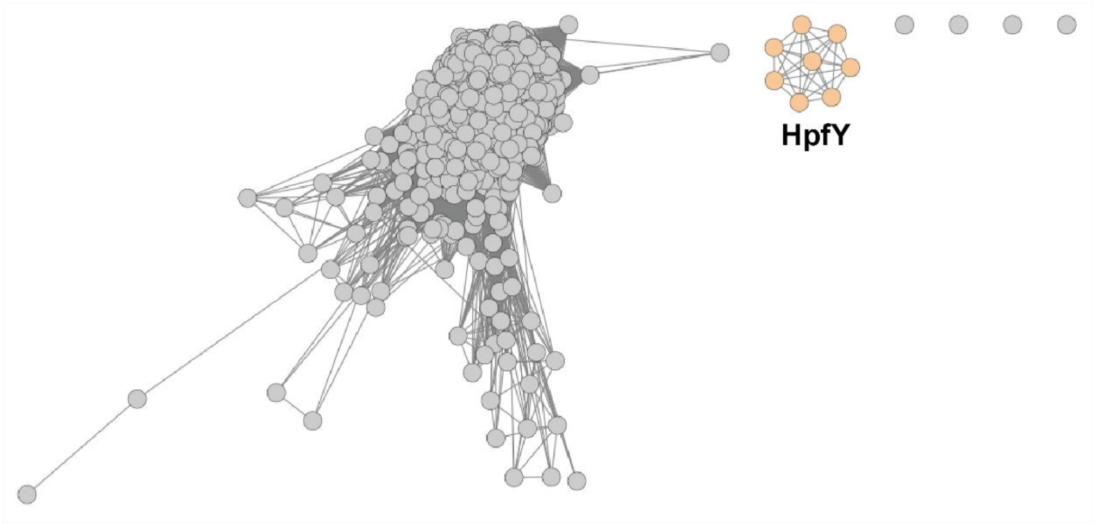

Figure S12. SSN for the CoA_ligase beta subunit homologs displayed at the E-value cut-off of 10^{-80} (Related to Figure 6). The most highly related proteins are grouped together in clusters, putatively sharing the same function. Each node represents a group of proteins sharing more than 80% sequence identity. HpfY Cluster is labelled in light orange.

Table S1. The protein information in the light orange cluster showing in Figure S12 (Related to Figure 6).

Accession number	Organism	Taxonomy
R2XLL0	*Enterococcus gilvus* ATCC BAA-350	1158614
A0A1Y4HVS1	*Collinsella* sp. An2	1965585
A0A0K9NA07	*Dorea* sp. D27	658665
C0BZV6	*Clostridium hylemonae* DSM 15053	553973
F0Z279	*Clostridium* sp. D5	556261
A0A4P5P8Q1	*Enterococcus florum*	2480627
A0A352UMH9	*Clostridiales bacterium*	1898207
R9JZX3	*Lachnospiraceae bacterium* M18-1	1235792
F0Z2A7	*Clostridium* sp. D5	556261
A0A1C5VQ00	uncultured *Ruminococcus* sp	165186
A0A395Y9C7	*Dorea* sp. AM58-8	2292346
A0A396KXH8	*Dorea* sp. AF36-15AT	2292041

Table S2. The abbreviations of proteins and substrates in this study (Related to Figure 1).

Abbreviations	Full name
SqvU	Sugar transportor
YihQ	Sulfoquinovosidase
SqvD	Sulfoquinovose isomerase
SqvA	6-Deoxy-6-sulfofructose transaldolase
YihU	Sulfolactaldehyde reductase
HpfG	Dihydroxypropanesulfonate dehydratase
HpfH	Activating enzyme for HpfG
HpfD	3-Sulfopropionaldehyde reductase
HpfE	3-Hydroxypropanesulfonate exporter
HpfX	3-Sulfopropionaldehyde dehydrogenase
HpfY	3-Sulfopropionate-CoA ligase subunit beta
HpfZ	3-Sulfopropionate-CoA ligase subunit alpha
TauE	Probable 3-Sulfopropionate exporter
SqwD	CoA-acylating sulfoacetaldehyde dehydrogenase
SqwKL	Sulfoacetate-CoA ligase
SlaB	Aldehyde dehydrogenase
SQ	Sulfoquinovose
SQGro	Sulfoquinovosyl glycerol
SF	6-Deoxy-6-sulfofructose
SLA	Sulfolactaldehyde
DHPS	Dihydroxypropanesulfonate
3-SPA	3-Sulfopropionaldehyde
3-HPS	3-Hydroxypropanesulfonate
3-SPC	3-Sulfopropionyl-CoA
3-SP	3-Sulfopropionate
SQDG	Sulfoquinovosyl diacylglycerol
G3P	Glyceraldehyde 3-phosphate
